# Characterization, localization and comparison of c-Kit+ lung cells in never smokers and smokers with and without COPD

**DOI:** 10.1186/s12890-018-0688-3

**Published:** 2018-07-31

**Authors:** Alejandra López-Giraldo, Tamara Cruz, Laureano Molins, Ángela Guirao, Adela Saco, Sandra Cuerpo, Josep Ramirez, Álvar Agustí, Rosa Faner

**Affiliations:** 1 Respiratory Institute, Hospital Clinic, University of Barcelona, Barcelona, Spain; 2 Institut d’investigacions Biomèdiques August Pi i Sunyer (IDIBAPS), Barcelona, Spain; 3 CIBER Enfermedades Respiratorias(CIBERES), Instituto de Salud Carlos III, Madrid, Spain; 4 Department of Pathology, Hospital Clinic, Barcelona, Spain; 5 Barcelona, Spain

**Keywords:** Bronchitis, Chronic obstructive pulmonary disease emphysema, Lung repair, Smoking, Lung stem cells

## Abstract

**Background:**

c-Kit + lung stem cells have been described in the human healthy lung. Their potential relation with smoking and/or chronic obstructive pulmonary disease (COPD) is unknown.

**Methods:**

We characterized and compared c-Kit+ cells in lung tissue of 12 never smokers (NS), 15 smokers with normal spirometry (S) and 44 COPD patients who required lung resectional surgery. Flow cytometry (FACS) was used to characterize c-Kit+ cells in fresh lung tissue disaggregates, and immunofluorescence (IF) for further characterization and to determine their location in OCT- embedded lung tissue.

**Results:**

We identified 4 c-Kit+ cell populations, with similar proportions in NS, S and COPD: *(1)* By FACS, c-Kit^high^/CD45+ cells (4.03 ± 2.97% (NS), 3.96 ± 5.30% (S), and 5.20 ± 3.44% (COPD)). By IF, these cells were tryptase+ (hence, mast cells) and located around the airways; *(2)* By IF, c-Kit^low^/CD45+/triptase- (0.07 ± 0.06 (NS), 0.03 ± 0.02 (S), and 0.06 ± 0.07 (COPD) cells/field), which likely correspond to innate lymphoid cells; *(3)* By FACS, c-Kit^low^/CD45-/CD34+ (0.95 ± 0.84% (NS), 1.14 ± 0.94% (S) and 0.95 ± 1.38% (COPD)). By IF these cells were c-Kit^low^/CD45-/CD31+, suggesting an endothelial lineage, and were predominantly located in the alveolar wall; and, *(4)* by FACS, an infrequent c-Kit^low^/CD45-/CD34- population (0.09 ± 0.14% (NS), 0.08 ± 0.09% (S) and 0.08 ± 0.11% (COPD)) compatible with a putative lung stem cell population. Yet, IF failed to detect them and we could not isolate or grow them, thus questioning the existence of c-Kit+ lung stem-cells.

**Conclusions:**

The adult human lung contains a mixture of c-Kit+ cells, unlikely to be lung stem cells, which are independent of smoking status and/or presence of COPD.

**Electronic supplementary material:**

The online version of this article (10.1186/s12890-018-0688-3) contains supplementary material, which is available to authorized users.

## Background

The mechanisms of lung repair and regeneration are not fully understood [1, 2]. A population of putative lung stem cells characterized by the surface expression of the c-Kit receptor (c-Kit+, also known as CD117) and the absence of hematopoietic, mesenchymal or epithelial cell markers, capable to repair the lung parenchyma in a cryoinjured mouse model has been described [3]. These results, however, have not been reproduced by other investigators who argued that this population of c-Kit+ cells might not have been adequately phenotyped and may in fact represent a population of endothelial progenitor cells [4–6] or even, mast cells, which share the c-Kit marker [7, 8].

Chronic obstructive pulmonary disease (COPD) is an important cause of morbidity and mortality worldwide [9]. Tobacco smoking is the main environmental risk factor for COPD, but not all smokers develop the disease [10]. We hypothesized that c-Kit+ lung dependent repair mechanisms may be deficient in smokers with COPD. To test this hypothesis we: (1) carefully characterized the phenotype of pulmonary cells expressing c-Kit; (2) located stem cells (c-Kit+CD45-) cells in the lung parenchyma; and (3) compared their number and location in never smokers and smokers with or without COPD.

## Methods

Methods are detailed in the on-line supplement.

### Study design and ethics

This observational, prospective and controlled study was approved by the Ethics Committee of our institution (ID 2012/7731). All participants signed their informed consent.

### Participants

We included 12 non-smokers, 15 smokers (> 10 pack/year) with normal spirometry and 44 smokers with COPD according to the GOLD criteria [10]. All of them required lung resectional surgery for diagnostic and/or therapeutic purposes due to early stage lung cancer or pulmonary solitary nodule. No participant received chemotherapy or radiotherapy before surgery or suffered from any other known chronic inflammatory condition.

### Lung function

Forced spirometry, static lung volumes and carbon monoxide diffusing capacity (DLCO) were determined in all participants (Medisoft, Surennes, Belgium). Reference values correspond to the Mediterranean population [11, 12]. The severity of airflow limitation was graded following GOLD recommendations [10].

### Tissue sampling & processing

Lung tissue was processed in less than 30 min after surgical extraction. After examination by a pathologist, non-tumoral affected tissue was selected; part was digested for flow cytometry (as described in the supplement) and the rest fixed in paraformaldehyde, embedded in OCT, frozen at − 50 °C in an isopentane bath and stored at − 80 °C until analysis.

### Flow cytometry

**Fig. 1:**
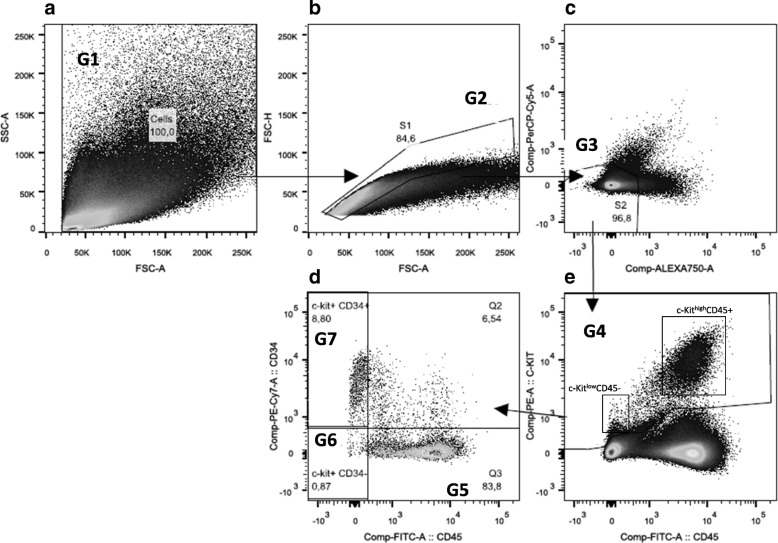
Gating strategy of C-Kit+ cells in flow cytometry: (**a**) all events were selected (G1); (**b**) cells aggregates were excluded (G2); (**c**) auto fluorescent cells were excluded (G3); (**d**) the expression of CD45 and CD34 was assessed in C-Kit+ cells identifying C-Kit+CD45 + CD34- (G5) C-Kit+CD45-CD34- cells (G6) and C-Kit+CD45-CD34+ cells (G7); and, finally (**e**) the c-Kit cell population is selected (G4). For further explanations, see text

Cells were stained as follows: (1) c-Kit determination tube: anti CD45-FITC anti C-Kit-PE and anti CD34- PECy7; (2) a C-Kit isotype control tube: anti CD45-FITC, anti IL-17A and anti CD34- PECy7; and (3) a negative control. Acquisition was done in BD FACS-CANTO II (BD, US) and analysis in Flow-Jo X software (LLC, US) following the gating strategy shown in Fig. 1.

### Immune-fluorescence

5 μm tissue slices were defrosted, rehydrated, subjected to antigen retrieval, permeabilised, washed and incubated overnight with primary antibodies: anti-CD117, anti-CD45, anti-tryptase, anti-CD31 (Additional file 1: Table S1). Specific staining was detected with secondary antibodies: Alexa Fluor 488/555 or 647 (Additional file 1: Table S1). Slices were mounted with prolong Gold with DAPI. Appropriate negative and cross reactivity controls were obtained (Additional file 1: Figure S1).

### Imaging analysis

Images were acquired using a TCS-SP5 laser scanning spectral confocal microscope (Leica Microsystems, Germany). A mosaic composition of consecutive and adjacent images of 1024 × 1024 pixels in 5 laser channels each was processed with the Matrix Screening software (Leica microsystems) that allows to visualize a representative tissue area that covered in all cases small airways, pulmonary vessels and *alveolar septae* (Additional file 1: Figure S2). Analysis of the tissue mosaics images was done using a customized macro of Image J software [13].

### Statistical analysis

Results are presented as n, proportion or mean ± SD, as appropriate. The Kruskal-Wallis test, followed by post-hoc Mann-Whitney contrast if needed, was used to compare continuous variables and Chi Square for discrete variables between groups. A *p* value < 0.05 was considered statistically significant.

## Results

Table 1 summarizes the main characteristics of the population studied. Briefly, the proportion of females was higher in non-smokers. Age and body mass index (BMI) were similar across groups. Cumulative tobacco smoking (pack-yr) was higher in COPD patients who had moderate airflow limitation whereas spirometry was normal in the other two groups. Additional file 1: Table S2 shows that these characteristics were similar in the subsample of the study population used for immune-fluorescence analysis.

**Table 1:** Characteristics (mean ± SD) of the individuals studied

	Non-smokers	Smokers	COPD	*P* value
	*N* = 12	*N* = 15	*N* = 44	
Age (years)	67.8 ± 9.3	61.4 ± 12.0	65.9 ± 7.6	0.18
Females/Males	10/2	5/10	9/35	0.0002
BMI (Kg/m^2^)	28.5 ± 6.6	27.7 ± 4.6	25.7 ± 3.9	0.41
Current/Former smokers	0/0	9/6	27/17	0.99
Cumulative smoking exposure (packs-year)	0 ± 0	36.3 ± 24.8	49.9 ± 20.1	0.01
FEV1/FVC (%)	77.9 ± 4.0	77.3 ± 7.5	59.5.0 ± 7.3	< 0.001
FEV1 (% reference)	97.5 ± 8.1	95.3 ± 9.4	75.0 ± 15.6	< 0.001

*BMI* Body Mass Index, *FEV1* Forced expiratory volume in 1 s, *FVC* Forced vital capacity

### Characterization of c-kit+ cells by flow cytometry

As shown graphically in Fig. 1e and quantitatively in Table 2, the most abundant FACS population of c-Kit+ cells in fresh lung tissue disaggregates in the three groups studied were c-Kit+^high^CD45+ cells. Differences between groups were not statistically significant (Additional file 1: Figure S3). Both mast cells and innate lymphoid cells (ILCs) co-express c-Kit and CD45 [14]. Additional file 1: Figure S4 shows that, by IF with tryptase co-staining the CD45 + c-Kit^high^ population represents mast cell, whereas ILCs are c-Kit^low^ CD45 + Triptase-.

**Table 2:** Percentage of C-Kit+ cells (in the population of live gated cells (G2)) determined by flow cytometry (mean ± SD)

% of gated cells	Non-smokers	Smokers	COPD	*P* value
	*N* = 12	*N* = 15	*N* = 44	
C-Kit^high^CD45+	4.03 ± 2.97	3.96 ± 5.30	5.20 ± 3.44	0.07
C-Kit^low^CD45-	1.05 ± 0.92	1.22 ± 1.01	1.04 ± 1.41	0.44
C-Kit^low^CD45-CD34+	0.95 ± 0.84	1.14 ± 0.94	0.95 ± 1.38	0.38
C-Kit^low^CD45-CD34-	0.09 ± 0.14	0.08 ± 0.09	0.08 ± 0.11	0.94

Around 22% of the c-Kit+ population by flow cytometry (i.e., 1% of total gated cells) was not of hematopoietic lineage (CD45-, Fig. 1e); of note, c-Kit intensity of this CD45- lineage was lower than that of CD45+ cells (CD45-, Fig. 1e). Their proportions were not different in the three groups studied (Table 2). In c-Kit+ CD45- cells, we analyzed the co-expression of CD34 and observed two different cell populations: (1) C-Kit^low^CD45-CD34+ cells (Fig. 1d. G6), which may represent a population of endothelial progenitor cells [15]; and, (2) C-Kit^low^CD45-CD34- (Fig. 1d. G5), that can correspond to a putative resident stem cell population [3]. Of note, this latter cell population corresponds to less than 0.1% of the total live-gated cells, and they did not appear as a well-defined population in the flow cytometer plot (Fig. 1d. G5). Table 2 shows that the proportion of these cell populations was not different across groups.

### Characterization of c-kit+ cell population by immunofluorescence (IF)

To localize the different lineages of c-Kit+ cells in lung tissue, we used triple IF staining containing c-Kit, CD45 and the mast cell marker tryptase, in a random subgroup of participants (Additional file 1: Table S2). In each of these patients we analyzed a mosaic composition of 169 consecutive images (× 40) covering a tissue area which included in all cases small airways, pulmonary vessels and alveolar *septae*. Additional file 1: Figure S2 is a representative image of the area studied per patient, and Additional file 1: Figure S4 an example of the staining of the three c-Kit different subpopulations identified.

As shown in Table 3, using IF the most abundant lung c-Kit+ cells were mast cells (c-Kit^high^CD45 + tryptase+). We also observed a less abundant subpopulation of c-Kit^low^CD45+ tryptase- cells (Table 3) that may represent ILCs [14]. Finally, less than 1% of c-Kit+ cells were negative for both mast cell markers (CD45 and tryptase), and their c-Kit staining intensity was lower than that of mast cells (c-Kit^low^CD45-tryptase-). To rule out the potential endothelial lineage of this latter c-Kit^low^CD45- tryptase- subpopulation, we evaluated if they also stained positive for CD31. We used this marker by IF instead of the CD34 used by FACS as in our hands the staining was able to better defined the cells. The c-Kit/CD31/CD45 was performed in a consecutive tissue sections to c-Kit/tryptase/CD45 and confocal images were obtained with additional 10 Z (axial) top to bottom slides. We found that all c-Kit^low^CD45- tryptase- cells stained positive for CD31 (Additional file 1: Figure S5), suggesting their endothelial cell lineage [15]. Additionally, we did not find co-staining of the stem cell markers Oct-4, NANOG, and KLF4 with c-Kit+ cells (Additional file 1: Figure S6). Thus we were not able to identify by IF the lung tissue stem cells defined as c-Kit^low^CD45- tryptase-CD31-. Finally, in agreement with FACS results, Table 3 shows that the number all the cell population identified by field was not different across groups.

**Table 3:** Number of C-Kit+ cells per field (mean ± SD) by immunofluorescence

C-Kit+ cells /field	Non-smokers	Smokers	COPD	*P* value
	*N* = 5	*N* = 5	*N* = 10	
C-Kit^high^CD45 + Tryptase+	5.25 ± 3.28	2.89 ± 0.65	3.72 ± 1.36	0.19
C-Kit^low^CD45-Tryptase-	0.07 ± 0.06	0.03 ± 0.02	0.06 ± 0.07	0.51
C-Kit^high^CD45 + Tryptase-	0.79 ± 0.47	0.48 ± 0.51	0.50 ± 0.32	0.37

### Location of c-kit+ cells in the lung parenchyma

**Fig. 2:**
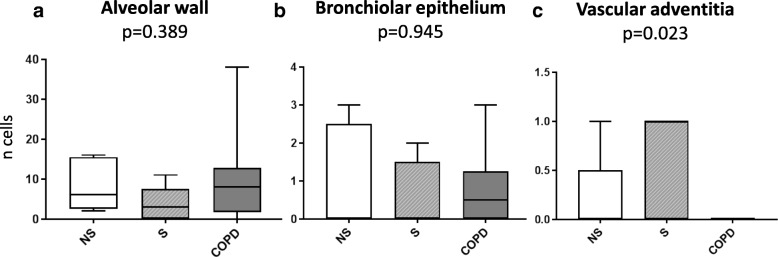
Box plot (median, 5-95% IC and SD (bars)) comparing the number of c-Kit^low^CD45-tryptase- cells (endothelial progenitors) in; panel (**a**) the alveolar wall, panel (**b**) bronchiolar epithelium, and panel (**c**) vascular adventitia in the three groups of subjects studied. Note the different Y range scales in the three different locations. For further explanations, see text

As expected, c-Kit^high^CD45 + tryptase+ (mast cells) were mainly located around the peribronchial intestitium. On the other hand, we observed that 89.0% of the c-Kit^low^CD45- tryptase- putative endothelial progenitor population was located in the alveolar wall, 8.9% in the bronchiolar epithelium, 1.6% in the vascular adventitia and 0.5% in the venous endothelium, without significant differences across groups (Fig. 2).

### Relationship with smoking status and lung function

We did not observe any correlation between smoking status or the severity of airflow limitation or DLCO impairment with the number of the different c-Kit+ populations investigated here (data not shown).

## Discussion

In 2011 Kajstura et al. reported the identification of a population of c-Kit+ putative stem cells in the human lung [3]. This publication generated both a great deal of interest and controversy [4–8]. To explore the role of c-Kit+ dependent repair mechanisms in COPD, we carefully characterized phenotypically all pulmonary cells expressing c-Kit, located them in the lung parenchyma and compared their number and location in never smokers and smokers with or without COPD. The two main and novel observations of our study were that, first, the human lung contains a heterogeneous mixture of, at least, four different c-Kit+ cell populations that likely include mast cells, innate lymphoid cells, endothelial progenitors and a putative but rare stem cell population; these observations clearly supports that c-Kit positivity cannot be used as “the” single stem cells marker [16]. And, second, that there were no significant differences in any c-Kit+ cell population studied here between never smokers and smokers with or without COPD, a hypothesis not previously tested to our knowledge.

### Characterization and location of lung c-kit+ cells

In keeping with some similar previous studies in cardiac tissue [4, 5, 7, 8, 17], we found that the adult human lung contains a heterogeneous mixture of distinct c-Kit+ cell populations: (1) the majority of them (85% by IF) are mast cells, since c-Kit^high^/CD45+ cells detected by FACS expressed the specific mast cell marker tryptase in IF [18], and were mainly located around the intestitium. The role of mast cells is well established in lung diseases associated with chronic inflammation [19] such as COPD [20] and asthma [21] and may contribute to their pathogenesis of bronchial remodeling [22]; (2) a smaller population (13% by IF) of c-Kit^low^CD45+ were tryptase- and may represent ILC [23], a family of innate immune cells that participate in the regulation of the immune response and tissue inflammation [24]. ILC lack specific antigen receptors and can produce several cytokines according to which they are classified in three groups (ILC1, ICL2, ILC3). ILC3 are known to express c-Kit in lung tissue [23]. (3) An even smaller population (1.6% by IF) of c-Kit ^low^CD45- that express CD34 + and/or CD31+ likely represent endothelial cell progenitors, which have been already described in lung tissue [15, 25]. In our study, they mostly located in the alveolar walls (Fig. 2); and, (4) finally, we found (only by FACS) a very small population (< 0.1% of gated cells) of c-Kit^low^CD45-CD34- cells that can potentially correspond to a potential putative lung stem cell population, as described by Kajstura et al.*...* [3] because they stained negative for cell linage markers. Yet, our IF analysis showed that c-Kit^low^ CD45-triptase- cells were positive for CD31, likely pinpointing towards an endothelial lineage. We were not able to identify a c-Kit^low^ lineage negative cells by IF.

In this context, some important differences between our study and that of Kajstura et al. [3] are worth mentioning. Firstly, they studied unused healthy young donor lungs whereas we obtained lung tissue samples from older patients requiring thoracic surgery for a variety of clinical reasons. Secondly, Kajstura et al reported high c-Kit staining intensity in lung stem cells [3] while in our study the bright c-Kit staining was only found in mast-cells, despite the fact we were used the c-Kit antibody from the same vendor. It is of note that, c-Kit is a receptor that is activated after binding its ligand, the stem cell factor (SCF). After binding SCF c-Kit receptors form homodimers that are internalized and degraded, so a low c-Kit expression (hence, intensity) is associated to an enhanced c-Kit consumption, thus cell activation [26]. Thirdly, Kajstura et al [3] reported that their c-Kit+ stem cells were mainly localized in the bronchioles whereas in this location we detected mast cells. Finally, our putative stem cell population (c-Kit^low^ CD45-CD34-) was detected by FACS only and we could not locate them in the lung parenchyma by IF. Unfortunately, we were not able to successfully sort and expand the c-Kit^low^CD45-CD34- cell population observed by FACS. In our hands the < 3000 cells obtained after sorting did not expanded and were not enough to perform functional assays to assess their potential stem cell characteristics. Future studies will have to use alternatives methodologies, such as clonal derivation [16], to explore this possibility and eventually clarify if this c-Kit^low^CD45-CD34- cells identified by FACS here really corresponds to a multipotent lung stem cell population.

### Effects of smoking and COPD

To our knowledge, this is the first study that compares the quantity and localization of c-Kit+ cells in never smokers and smokers with and without COPD. We did not find differences between them. Likewise, we did not identify any significant relationship with physiological measures (severity of airflow limitation, DLCO) or smoking status. Hence, these results do not support our working hypothesis that c-Kit+ stem cells may be different in number and/or location in smokers with and without COPD. Yet, because we could not perform in vitro functional assays in this cell population, we cannot exclude that the function of these c-Kit+ cells may be different in these groups. In any case, these results contribute to delineate more precisely the quantity, type, localization and relationship with smoking and COPD of c-Kit+ cells in the adult human lung.

### Strengths and limitations

Our study has a number of strengths and limitations. Among the former, the relative large number of participants included in the study, its controlled design, the use of combined FACS to characterize phenotypically these c-Kit+ cells and IF to locate them, as well as the comparison of smokers with and without COPD are strengths of our paper. Among the latter that, we acknowledge that the quantitation of putative stem cells in the context of very low cell numbers is very challenging so ours should be considered only as indicative data. Likewise, for this same reason, we could not isolate, expand in culture and perform functional assays in the putative stem cells population identified by FACS. Also, due to its low percentage, the failure to conform a well-defined population in forward side plots and the latter failure to detect them by IF we cannot exclude the possibility that these events are an artifact of the detection technique. In any case, however, our results cast serious doubts about the existence of c-Kit+ lung stem cells in humans.

## Conclusions

This study shows that the adult human lung contains a heterogeneous population of c-Kit expressing cells, including mast cells, innate lymphoid cells and putative endothelial cell progenitors. Only using FACS we were able to identify < 0.1% cells meeting the cell-surface criterion of c-Kit+ stem cells, but we could not verify their presence by IF or functional analyses. All in all, these results question seriously the existence of c-Kit+ lung stem cells in humans. Finally, contrary to our original hypothesis, we failed to identify significant differences in c-Kit+ cells between smokers with and without COPD.

## Additional file

Additional file 1: **Figure S1.** Absence of cross reactivity between host species in primary and secondary antibodies. **Figure S2.** Representative image of a lung tissue mosaic. **Figure S3.** Percentage of c-Kit+/CD45+ gated cells by flow cytometry in the three study groups. **Figure S4.** C -kit+ cell populations in lung tissue. **Figure S5.** Representative image showing that C-kit^low^CD45- cells determined by IF stain positively for CD31. **Figure S6.** Representative images showing C-Kit+ cells with stem cells markers. **Table S1.** Primary and secondary antibodies for immune-histochemistry staining. **Table S2.** Clinical characteristics of the subpopulaton included in the immunofluorescence analysis (mean ± SD). (ZIP 1150 kb)

## Abbreviations

COPD: Chronic obstructive pulmonary disease
DLCO: Carbon monoxide diffusing capacity
FACS: Flow cytometry
IF: Immunofluorescence
NS: Never smokers
S: Smokers with normal spirometry
SCF: Stem cell factor
