# Supplementary material for: Characterization, localization and comparison of c-Kit+ lung cells in never smokers and smokers with and without COPD
Source: BMC Pulm Med. 2018 Jul 31;18:123. doi: 10.1186/s12890-018-0688-3 (PMC6066937; doi:10.1186/s12890-018-0688-3)
Supplement: Supplementary file 1 — Figure S1. Absence of cross reactivity between host species in primary and secondary antibodies. Figure S2. Representative image of a lung tissue mosaic. Figure S3. Percentage of c-Kit+/CD45+ gated cells by flow cytometry in the three study groups. Figure S4. C -kit+ cell populations in lung tissue. Figure S5. Representative image showing that C-kitlowCD45- cells determined by IF stain positively for CD31. Figure S6. Representative images showing C-Kit+ cells with stem cells markers. Table S1. Primary and secondary antibodies for immune-histochemistry staining. Table S2. Clinical characteristics of the subpopulaton included in the immunofluorescence analysis (mean ± SD). (ZIP 1150 kb) [file 12890_2018_688_MOESM1_ESM.zip › On_line_Lopez-Giraldo Lung ckit+ cells June 14 2018_ cleanR3.docx]

16 June 2018

Supplementary Information for:

**CHARACTERIZATION, LOCALIZATION AND COMPARISON OF C-KIT+ LUNG CELLS IN NEVER SMOKERS AND SMOKERS WITH AND WITHOUT COPD**

Alejandra López-Giraldo^1,2,3^, Tamara Cruz^2,3^, Laureano Molins^1-2^, Ángela Guirao^1,2^, Adela Saco^2,4^, Sandra Cuerpo^1,2,3^, Josep Ramirez^2,4^,

Álvar Agustí^1,2,3^, Rosa Faner^2,3^

1. Respiratory Institute, Hospital Clinic, University of Barcelona, Spain.
2. Institut d'investigacions Biomèdiques August Pi i Sunyer (IDIBAPS), Barcelona, Spain.
3. CIBER Enfermedades Respiratorias(CIBERES), Instituto de Salud Carlos III, Spain.
4. Department of Pathology, Hospital Clinic, Barcelona, Spain.

**On-line supplement:** Word count: 1,238; Tables, 2; Figures: 6.

**Methods**

**Lung tissue processing**

Fresh lung tissue was collected during surgery and processed in the next 30 minutes. After examination by a pathologist, non-cancer affected tissue was weighted, washed in PBS buffer (Gibco, Life technologies, US) to remove blood excess, cut into small pieces and digested enzymatically with 0,5mg/ml collagenase P (Roche, Mannheim, Germany) and 0,1 mg/ml DNase I (Roche, Mannheim, Germany) during 30 minutes at 37ºC. Tissue was then mechanically digested with the GentleMACS system, following manufacturer instructions (Miltenyi Biotec, Germany). A maximum of 2 gr. of tissue per GentleMACS C tube were processed. Homogenized tissue was then filtered with a 100μm cell strainer (BD) to eliminate undigested tissue. Erythrocytes were lysed with hemolysis buffer (8,3g NH_4_Cl, 1 g NaHCO_3_ and 0,04g of disodium EDTA in 1l distillate water and sterilized by filtration). Finally, additional filtration though 70 μm and 40 μm cell strainer (BD) was done.

**Flow cytometry**

Cells were re-suspended in PBS, counted and volume adjusted with PBS at a concentration of 30x10^6^ cells/ml. Three tubes with 100 µl of cell suspension were then stained as follows: *(1)* a C-kit determination tube with 10 µl of anti CD45-FITC (BD 555482), 10 µl of anti Ckit-PE (DAKO R7145) and 5 µl of anti CD34- PECy7 (BD 348811); *(2)* a c-kit isotype control tube with 10 µl of CD45-FITC (BD 555482), 10 µl of anti-IL-17A (Miltenyi Biotech, 130091521) and 5 µl of CD34- PECy7 (BD, 348811); and *(3)* a not-stained negative control. Tubes were incubated during 30 minutes at 4ºC in the dark, washed with PBS, centrifuged at 400G during 6 minutes, re-suspended in PBS with 10% fixative (Immunochemistry technologies, US) and analyzed in BD FACS-CANTO II (BD, US). For each tube ≥10^6^ cells were analyzed using Flow-Jo X software (LLC, US), following the gating strategy shown in Figure 1: *(1)* cells aggregates were excluded using FCS-H and SSC-A(G2); *(2)* auto fluorescent cells were excluded with two empty channels; (G3) we selected the population of the C-kit+ cells, in this case the gate was set up with an isotype control, that was included in every experiment (G4); and, finally *(3)* the expression of CD45 and CD34 was assessed in C-kit+ cells identifying C-kit+CD45-CD34- cells (G5) and C-kit+CD45-CD34+ cells (G6).

To set up the flow cytometry methodology, we tested several concentrations of collagenase and DNAse and selected the minimum concentration that yielded similar amounts of cells CD45 cells. Likewise, we disaggregated mechanically two lung tissue samples to prove that this enzymatic treatment did not affect the epitopes that we were assessing by FACS.

**Lung Immunofluorescence**

A representative portion of the tissue sample was fixed with 4% paraformaldehyde during 24h at 4°C, then embedded in OCT (Sakura, Fisher Technologies) and frozen at -50°C in an isopentane bath and stored at -80°C. Cryostat slices (5µm) were obtained and stored at -80ºC until staining.

To stain them, they were defrosted at room temperature and allowed to dry at least for 2 hours. Then, they were rehydrated with PBS and subjected to antigen retrieval with 1% SDS solution, permeabilized with 0,5% trito-X100, blocked with 1% bovine serum albumin-10X (GIBCO, Life Technologies, US) and 10% normal donkey serum (Merck, S30-100ML), washed and incubated overnight at 4°C in the a dark chamber with the corresponded concentration of the primary antibodies diluted in antibody diluent with background reducing components (DAKO, S30022) and 10% of normal donkey serum: anti-CD117 (DAKO A4502), anti-CD45 (Novus Biologicals YTH24.5), anti-tryptase (DAKO, M7052), anti-CD31 (DAKO, M0823) (Table S2). Tissue sections were then washed three times with PBS and the corresponding secondary antibodies were added for 1 hour at room temperature in a dark chamber: Alexa Fluor 488 (Molecular Probes), Alexa Fluor 555 (Molecular Probes) and Alexa Fluor 647 (Molecular Probes) (Table S2). Tissue slices were washed three times with PBS and then mounted with prolong Gold with DAPI for nuclei staining (Molecular Probes, P36935). Negative controls were done applying only the secondary antibody to control for unspecific staining. Absence of cross reactivity between antibodies host species was also verified.

(Supplement Figure 1). The staining with Oct4, NANOG and KLF4 was done in the same conditions, with the addition of 0.5% Triton to the permeabilization buffer. Positive controls for Oct-4, NANOG and KLF4 were made in human embrionic stem cells.

**Microscope imaging and analysis**

Images were acquired using a TCS-SP5 laser scanning spectral confocal microscope (Leica Microsystems, Germany), at the Advanced Optical Microscopy Unit from the University of Barcelona, using a 40X- oil immersion objective with 1X digital zoom and standard pinhole. A mosaic composition of consecutive and adjacent images of 1024x1024 pixels in 5 laser channels each, was processed with the Matrix Screening software (Leica microsystems) that allows to visualize a representative tissue area that covered in all cases small airways, pulmonary vessels and alveolar septae (Figure 2). For the C-kit-, CD45 and tryptase triple staining, the tissue mosaic consisted in 169 images (13 by 13 images) and for the C-kit, CD45, CD31 triple staining, of 36 images (6 by 6 images). Additional 10 axial (Z) slides were obtained from top to bottom of the tissue section in order to assess several layers of the sample.

Analysis of the tissue mosaics images was done using a customized macro of Image J software (14) (National Institute of Health). Briefly, from the 5 channels Matrix Screener mosaic, 1 stack per channel was obtained with single images of 1024x2024 pixels. Then, positive cells for C-kit staining (555 channel stack) were segmented and identified as regions of interest (ROIs). The ROIs were then manually verified in order to fulfill the criteria of membrane and/or cytoplasmic C-kit staining. For all ROIs, the area and mean intensity were measured in each stack. In the C-kit, CD45 and CD31 triple staining, and additional hyperstack from the 5 channels and 10 Z slices was created followed by the same procedure of ROIs segmentation and verification. Measurement of the intensity of Sum Projection over all fluorescent channels was determined. Mean intensity of a background area in each tissue mosaic was also quantified to define a threshold for positive or negative staining. For each ROI, a first observer (ALG) determined the presence of positive staining in each stack, based on the threshold limit of the background and the mean intensity of the specific staining, and then, determined a positive (high/low) or negative staining for each antibody, followed by a second observer analysis (TC). The localization of C-kit+ cells in the mosaic tissue was determined by an experienced lung pathologist (AS).

**Table S1.** Primary and secondary antibodies for immune-histochemistry staining.

| **Primary antibody**  **Brand, reference and concentration** | **Secondary antibody**  **Brand, reference and concentration** |
| --- | --- |
| Anti-CD117 Policlonal rabbit anti-human.  DAKO, A4502  1:300 μl | Alexa Fluor 555 IgG donkey anti-rabbit  Life Technologies, A31572  1:200 μl |
| Anti CD45 IgG2b rat anti-human  Novus Biologicals YTH24.5  1:100 μl | Alexa Fluor 488 IgG donkey anti-Rat  Molecular Probes A21208  1:200 μl |
| Anti-tryptase IgG1kappa mouse anti-human  DAKO M7052  1:200 μl | Alexa Fluor 647 IgG donkey anti-mouse  Molecular Probes A 31571  1:200 μl |
| Anti-CD31 IgG1 mouse anti-human  DAKO, M0823  1:100 μl | Alexa Fluor 647 IgG donkey anti-mouse  Molecular Probes A 31571  1:200 μl |
| Anti-Oct 4 IgG1 mouse anti-human  Millipore, MABA4401  1:150 μl | Alexa Fluor 647 IgG donkey anti-mouse  Molecular Probes A 31571  1:200 μl |
| Anti Nanog IgG2aƙ mouse anti-human  Millipore, MABD24  1:300 μl | Alexa Fluor 647 IgG donkey anti-mouse  Molecular Probes A 31571  1:200 μl |
| Anti-KLF 4 IgG1mouse anti-human  Millipore, MABC631  1:50 μl | Alexa Fluor 647 IgG donkey anti-mouse  Molecular Probes A 31571  1:200 μl |

**Table S2.** Clinical characteristics of the subpopulaton included in the immunofluorescence analysis (mean±SD).

|  | **Non- smokers** | **Smokers** | **COPD** | **P value** |
| --- | --- | --- | --- | --- |
| Number of patients | 5 | 5 | 10 |  |
| Age (years) | 70.2±13.0 | 63.0±10.7 | 62.4±8.5 | 0.34 |
| Females/Males | 4/1 | 2/3 | 4/6 |  |
| BMI (Kg/m^2^) | 27.1±5.1 | 30.9±5.6 | 25.0±3.9 | 0.13 |
| Current smokers | NA | 4 | 6 |  |
| Former smokers | NA | 1 | 4 |  |
| Cumulative smoking exposure (packs-year) | NA | 44.0±32.0 | 45.4±19.3 | 0.46 |
| FEV1/FVC (%) | 76.7±5.5 | 75.8±2.7 | 60.4±7.6 | <0.001 |
| FEV1 (%reference) | 94.6±7.0 | 96.2±8.5 | 80.4±14.9 | 0.05 |

BMI: Body Mass Index. FEV1: Forced expiratory volume in 1 second. FVC: Forced vital capacity. NA: do not apply.

**SUPPLEMENTARY FIGURE LEGENDS**

**Figure S1**. Absence of cross reactivity between host species in primary and secondary antibodies. Panel A: Primary antibody: C- Kit (Rabbit anti Human) vs Secondary antibody: Alexa Fluor 488 (Donkey anti Rat). Panel B: Primary antibody: CD45 (Rat anti Human) vs Secondary antibody: Alexa Fluor 488 (Donkey anti Rat). Panel C: Primary antibody: C- Kit (Rabbit anti Human) vs Secondary antibody: Alexa Fluor 555 (Donkey anti Rabbit). Panel D: Primary antibody: CD45 (Rat anti Human) vs Secondary antibody: Alexa Fluor 555 (Donkey anti Rabbit). For further explanations, see text.

**Figure S2**. Representative image of a lung tissue mosaic. In each of the samples analyzed by IF a mosaic of 13x13 images with the 40x objective (total = 169 images, 1024x1024 pixels, in the figure each square is an image) were acquired with the SPS5 Confocal microscope and the Matrix Screener Software. Blue staining is DAPI, c-kit in green, CD45 in red and tryptase in magenta. For further details, see text.

**Figure S3**. Percentage of c-Kit+/CD45+ gated cells by flow cytometry in the three study groups. For further explanations, see text.

**Figure S4**. C -kit+ cell populations in lung tissue. Representative images of the three populations determined by IF. Panel A) C-kit^high^ CD45+ Tryptase+. Panel B)

C-kitlow CD45+ Tryptase-. Panel C) C-kitlow CD45- Tryptase-. The C-kit staining is displayed in green, CD45 in red, and Tryptase in magenta, the forth column represents the merged image. For further explanations, see text.

**Figure S5**. Representative image showing that C-kit^low^CD45- cells determined by IF stain positively for CD31. Panel A) the white arrow points to a C-kit^low^CD45-cell that in Panel B) is shown to express CD31. The C-kit staining is displayed in green, CD45 in red, and CD31 in magenta. For further explanations, see text.

**Figure S6.** Representative images showing C-Kit+ cells with stem cells markers: NANOG (Panel A), Oct-4 (Panel B), KLF4 (Panel C). There is no intra-nuclear staining of these markers in C-Kit+ cells.
