# Supplementary figures and images for: Characterization, localization and comparison of c-Kit+ lung cells in never smokers and smokers with and without COPD
Source: BMC Pulm Med. 2018 Jul 31;18:123. doi: 10.1186/s12890-018-0688-3 (PMC6066937; doi:10.1186/s12890-018-0688-3)

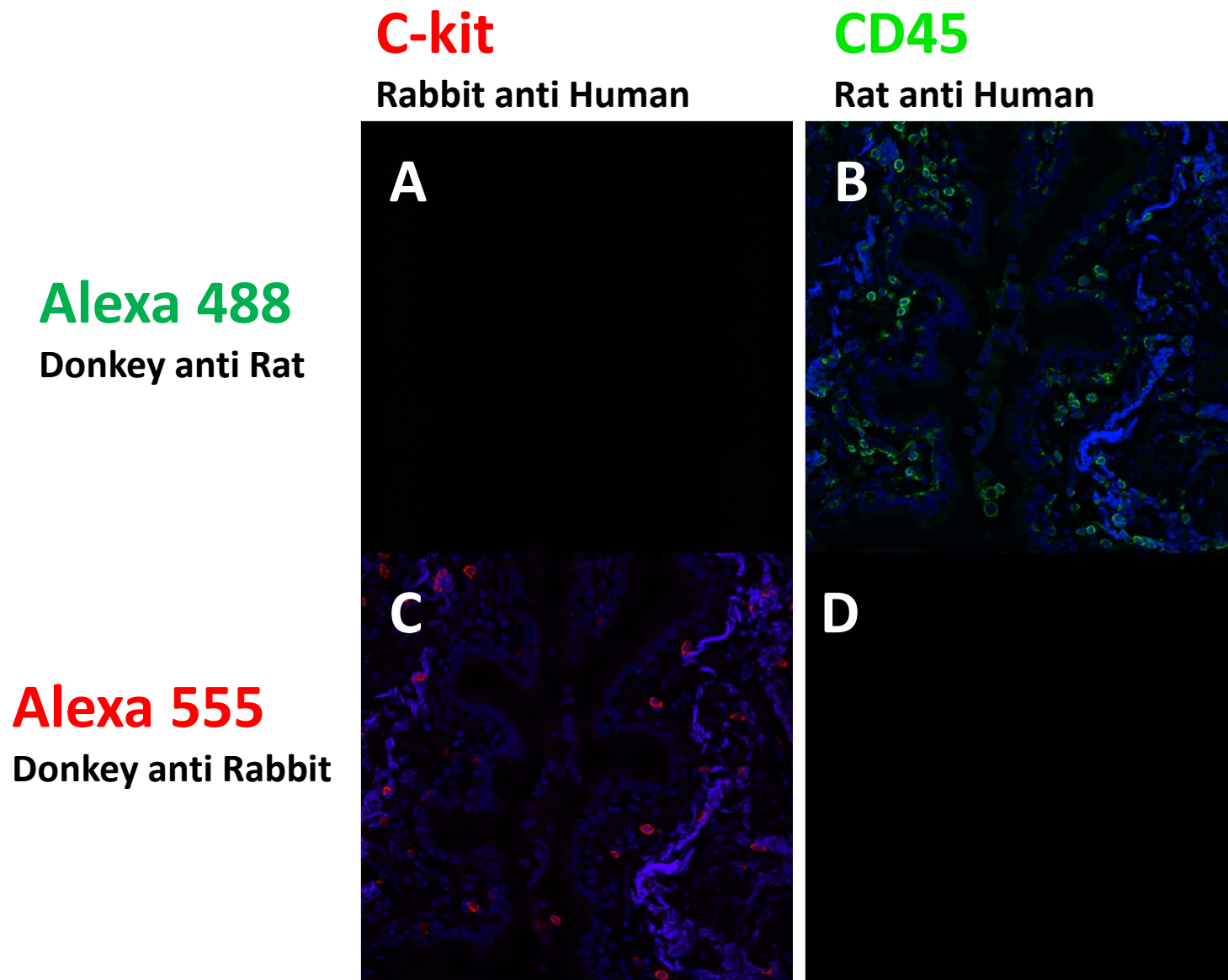

Figure S1

Supplement: Supplementary file 1 — Figure S1. Absence of cross reactivity between host species in primary and secondary antibodies. Figure S2. Representative image of a lung tissue mosaic. Figure S3. Percentage of c-Kit+/CD45+ gated cells by flow cytometry in the three study groups. Figure S4. C -kit+ cell populations in lung tissue. Figure S5. Representative image showing that C-kitlowCD45- cells determined by IF stain positively for CD31. Figure S6. Representative images showing C-Kit+ cells with stem cells markers. Table S1. Primary and secondary antibodies for immune-histochemistry staining. Table S2. Clinical characteristics of the subpopulaton included in the immunofluorescence analysis (mean ± SD). (ZIP 1150 kb) [file 12890_2018_688_MOESM1_ESM.zip › Figure S1R3.pdf]

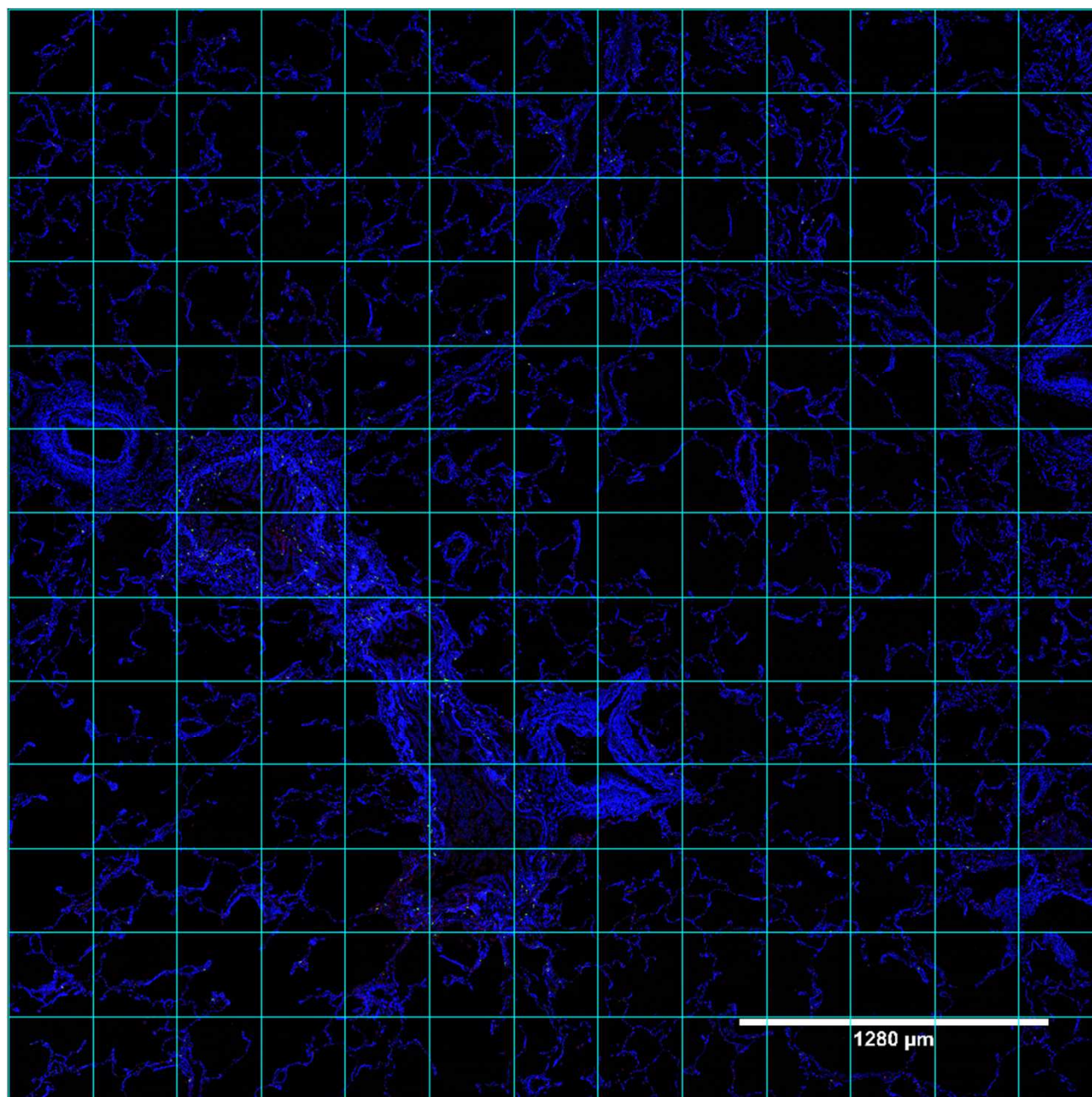

Mosaic 13x13 images  
Total 169 images  
1024x1024 pixels  
40X  
Software Matrix Screener  
Confocal microscope

Figure S2

Supplement: Supplementary file 1 — Figure S1. Absence of cross reactivity between host species in primary and secondary antibodies. Figure S2. Representative image of a lung tissue mosaic. Figure S3. Percentage of c-Kit+/CD45+ gated cells by flow cytometry in the three study groups. Figure S4. C -kit+ cell populations in lung tissue. Figure S5. Representative image showing that C-kitlowCD45- cells determined by IF stain positively for CD31. Figure S6. Representative images showing C-Kit+ cells with stem cells markers. Table S1. Primary and secondary antibodies for immune-histochemistry staining. Table S2. Clinical characteristics of the subpopulaton included in the immunofluorescence analysis (mean ± SD). (ZIP 1150 kb) [file 12890_2018_688_MOESM1_ESM.zip › Figure S2R3.pdf]

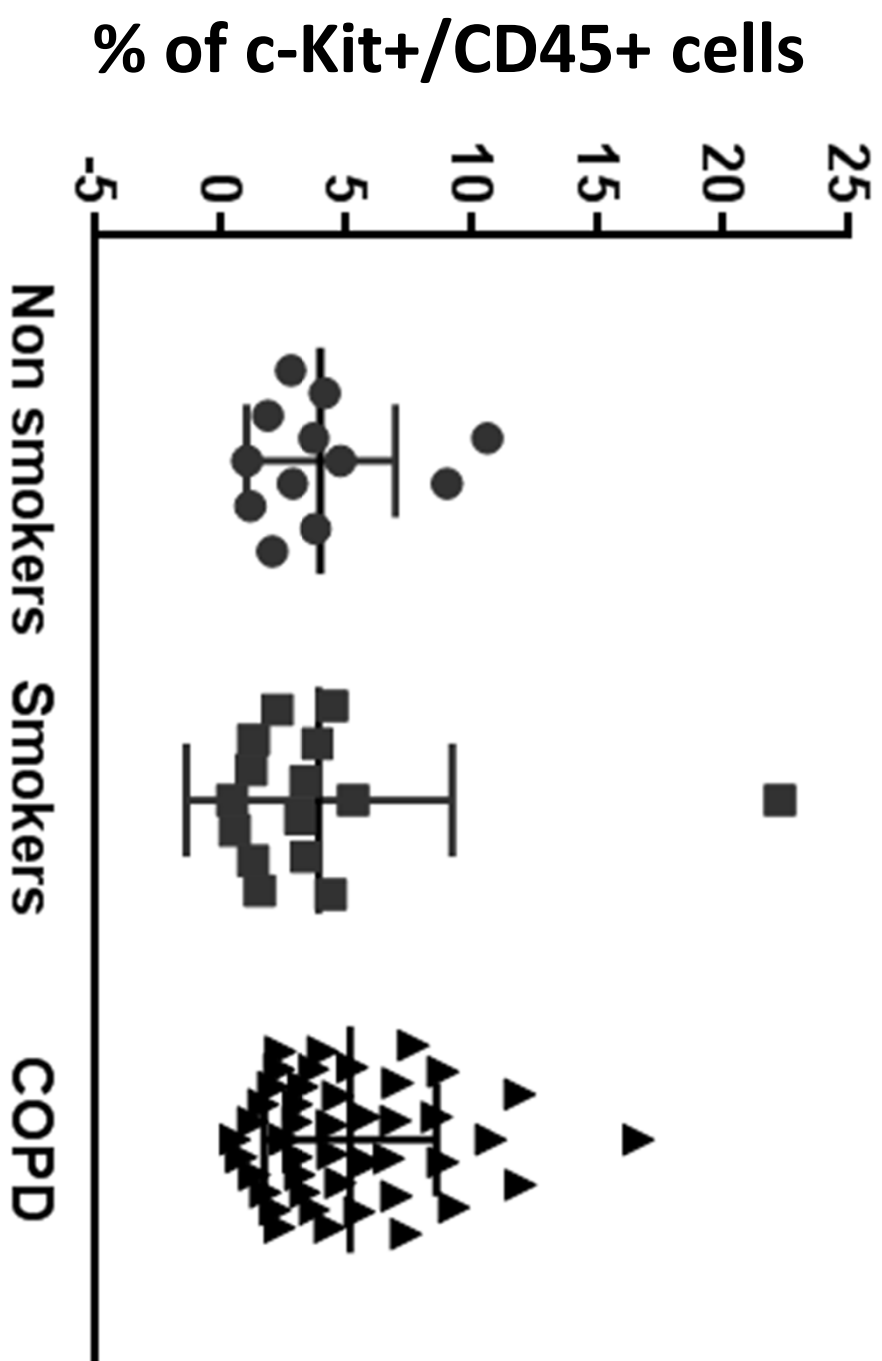

Figure S3

Supplement: Supplementary file 1 — Figure S1. Absence of cross reactivity between host species in primary and secondary antibodies. Figure S2. Representative image of a lung tissue mosaic. Figure S3. Percentage of c-Kit+/CD45+ gated cells by flow cytometry in the three study groups. Figure S4. C -kit+ cell populations in lung tissue. Figure S5. Representative image showing that C-kitlowCD45- cells determined by IF stain positively for CD31. Figure S6. Representative images showing C-Kit+ cells with stem cells markers. Table S1. Primary and secondary antibodies for immune-histochemistry staining. Table S2. Clinical characteristics of the subpopulaton included in the immunofluorescence analysis (mean ± SD). (ZIP 1150 kb) [file 12890_2018_688_MOESM1_ESM.zip › Figure S3R3.pdf]

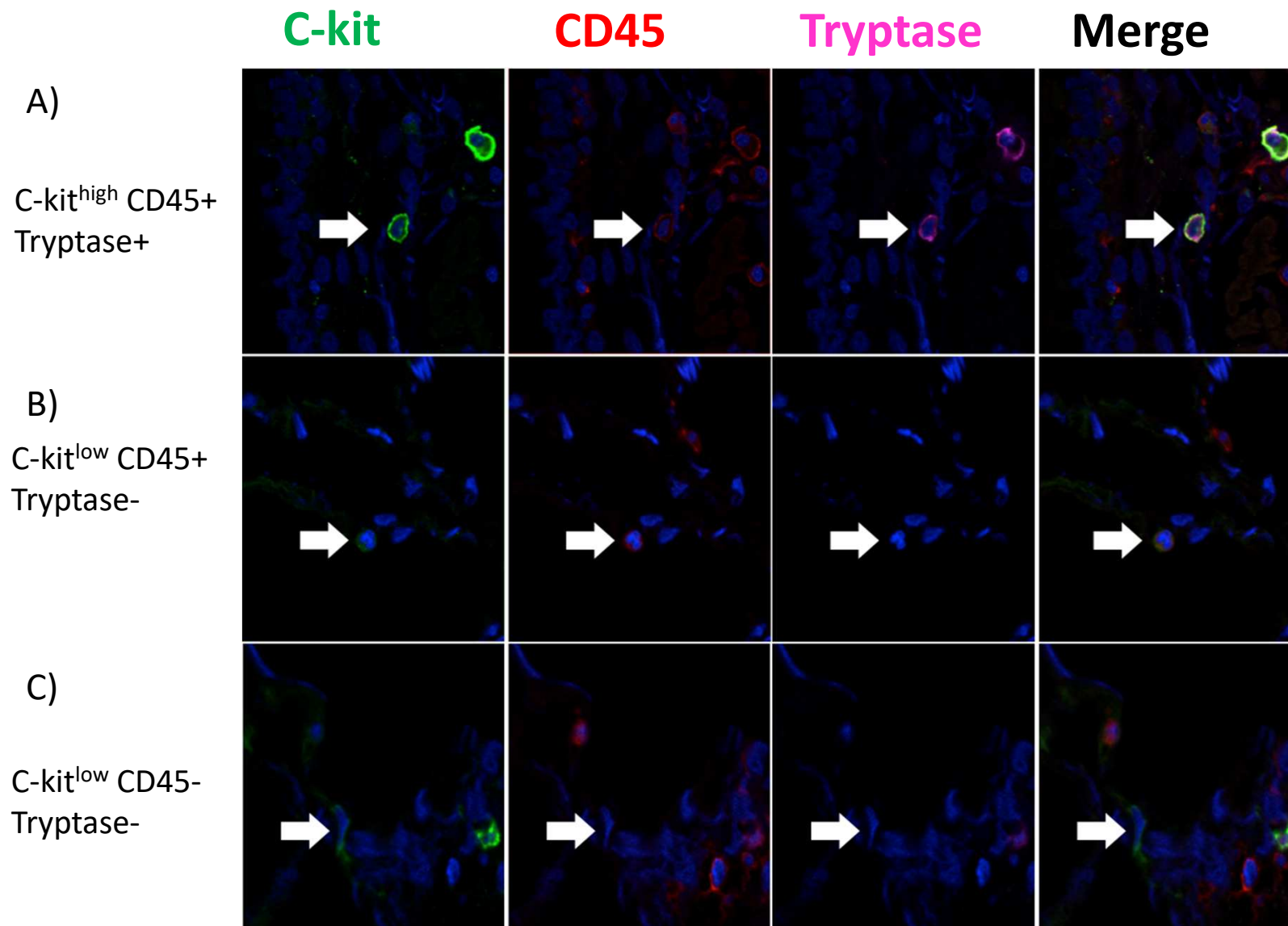

Figure S4

Supplement: Supplementary file 1 — Figure S1. Absence of cross reactivity between host species in primary and secondary antibodies. Figure S2. Representative image of a lung tissue mosaic. Figure S3. Percentage of c-Kit+/CD45+ gated cells by flow cytometry in the three study groups. Figure S4. C -kit+ cell populations in lung tissue. Figure S5. Representative image showing that C-kitlowCD45- cells determined by IF stain positively for CD31. Figure S6. Representative images showing C-Kit+ cells with stem cells markers. Table S1. Primary and secondary antibodies for immune-histochemistry staining. Table S2. Clinical characteristics of the subpopulaton included in the immunofluorescence analysis (mean ± SD). (ZIP 1150 kb) [file 12890_2018_688_MOESM1_ESM.zip › Figure S4R3.pdf]

A)

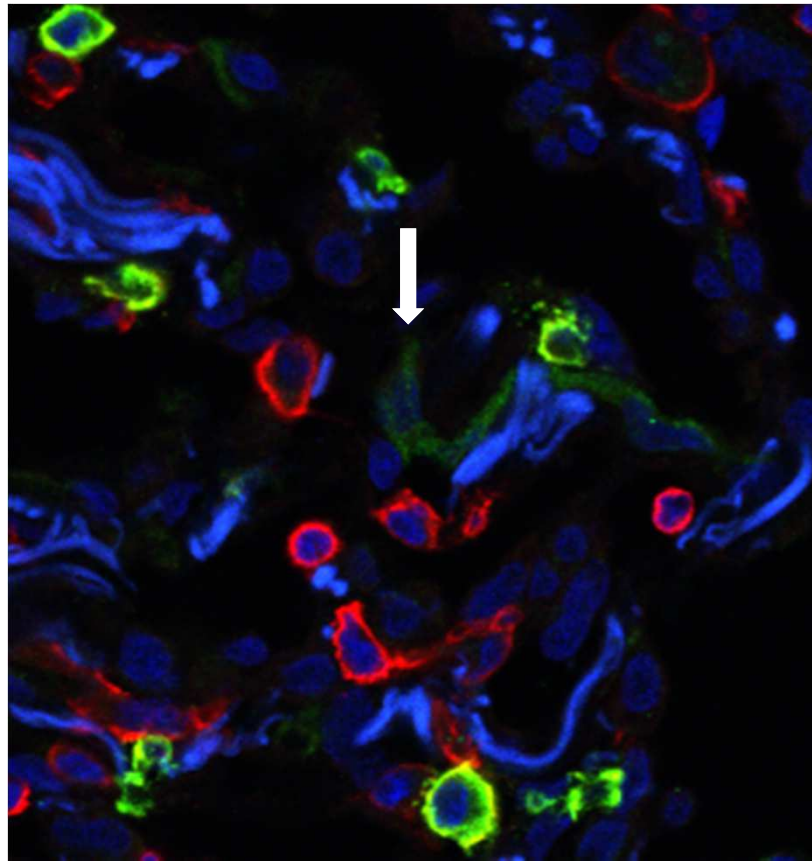

C+kit CD45

B)

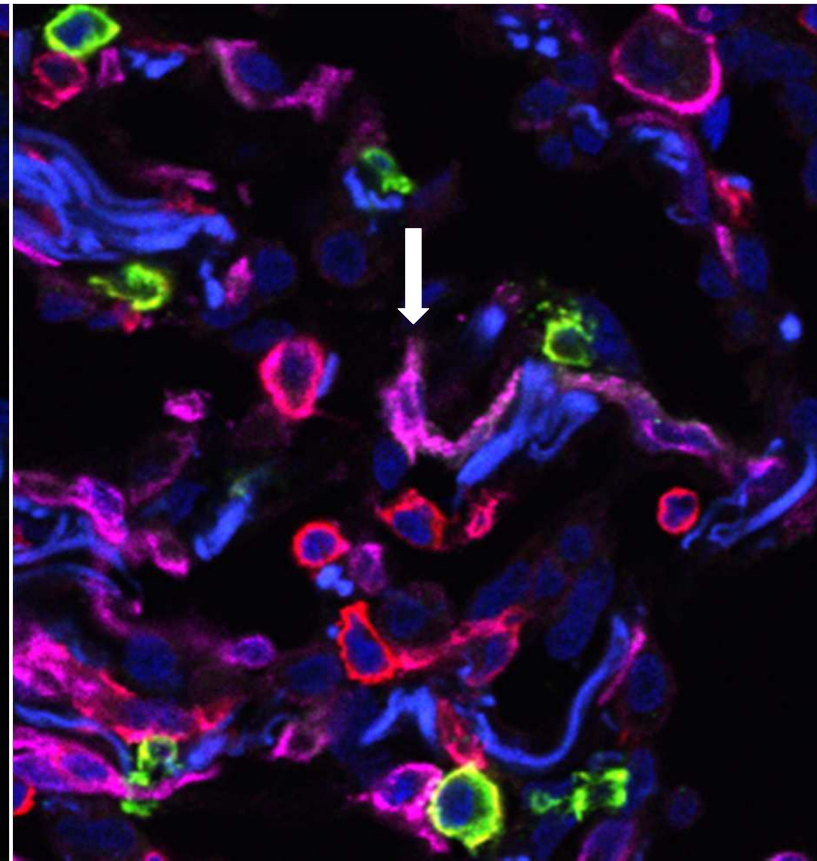

C+kit CD45 CD31

Figure S5

Supplement: Supplementary file 1 — Figure S1. Absence of cross reactivity between host species in primary and secondary antibodies. Figure S2. Representative image of a lung tissue mosaic. Figure S3. Percentage of c-Kit+/CD45+ gated cells by flow cytometry in the three study groups. Figure S4. C -kit+ cell populations in lung tissue. Figure S5. Representative image showing that C-kitlowCD45- cells determined by IF stain positively for CD31. Figure S6. Representative images showing C-Kit+ cells with stem cells markers. Table S1. Primary and secondary antibodies for immune-histochemistry staining. Table S2. Clinical characteristics of the subpopulaton included in the immunofluorescence analysis (mean ± SD). (ZIP 1150 kb) [file 12890_2018_688_MOESM1_ESM.zip › Figure S5R3.pdf]

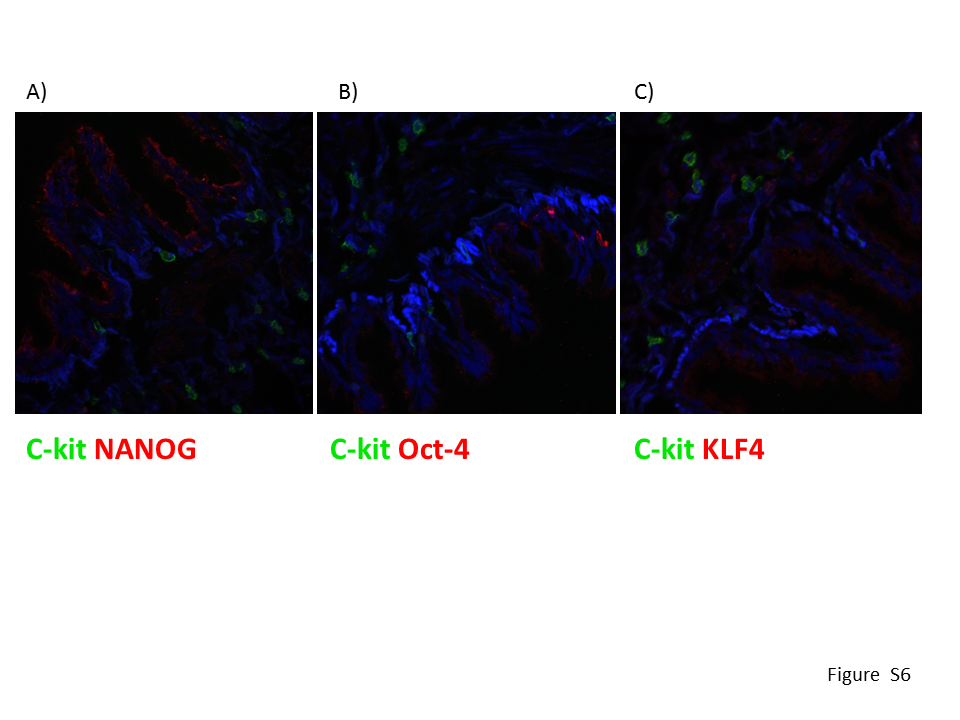

Supplement: Supplementary file 1 — Figure S1. Absence of cross reactivity between host species in primary and secondary antibodies. Figure S2. Representative image of a lung tissue mosaic. Figure S3. Percentage of c-Kit+/CD45+ gated cells by flow cytometry in the three study groups. Figure S4. C -kit+ cell populations in lung tissue. Figure S5. Representative image showing that C-kitlowCD45- cells determined by IF stain positively for CD31. Figure S6. Representative images showing C-Kit+ cells with stem cells markers. Table S1. Primary and secondary antibodies for immune-histochemistry staining. Table S2. Clinical characteristics of the subpopulaton included in the immunofluorescence analysis (mean ± SD). (ZIP 1150 kb) [file 12890_2018_688_MOESM1_ESM.zip › Figure S6R3.TIF]
